# Effects of TGMXD-208, a Novel PI3K Inhibitor, on Adjuvant-induced Arthritis Rats by Suppressing PI3K/AKT Signaling Pathway

**DOI:** 10.2174/0118715303373717250509063328

**Published:** 2025-05-22

**Authors:** Jiahua Yin, Jiayu Wang, Wenrui Su, Ran Tang, Zhifang Qin, Xiaoyi Jia, Xiaodong Ma, Shuangying Gui

**Affiliations:** 1 School of Pharmacy, Anhui University of Chinese Medicine, Hefei, Anhui 230012, China;; 2 Anhui Province Key Laboratory of Research & Development of Chinese Medicine, Hefei, Anhui 230012, China;; 3 Institute of Medicinal Chemistry, Anhui Academy of Chinese Medicine, Hefei, Anhui 230012, China;; 4 Institute of Pharmaceutics, Anhui Academy of Chinese Medicine, Hefei, Anhui 230012, China

**Keywords:** PI3K inhibitor, TGMXD-208, adjuvant-induced arthritis, PI3K/AKT signaling pathway, rheumatoid arthritis, vascular opacities

## Abstract

**Background:**

The PI3K/AKT signaling pathway is critical for immune cell proliferation, differentiation, survival, and inflammatory cytokine release. TGMXD-208, a novel dual PI3K inhibitor, selectively targets both PI3Kδ and PI3Kγ, positioning it as a potential therapeutic agent for Rheumatoid Arthritis (RA).

**Methods:**

This study evaluated ankle joint swelling and arthritis index and employed HE staining, X-ray imaging, and small animal ultrasonography to assess rat joint tissues. Serum antibodies for IL-1β, IL-6, and TNF-α were determined, as well as organ indices for the spleen and thymus. Western blotting and immunohistochemistry were used to measure the expression and phosphorylation of key enzymes in the PI3K/AKT pathway. The study also examined the impact of TGMXD-208 on hematological parameters and organs.

**Results:**

Our investigation found that TGMXD-208 dramatically reduced ankle redness and swelling, as well as the arthritis index, in rats in the model group. Additionally, TGMXD-208 reduced arthralgia, deformity, and synovial hyperplasia of the knee joint and accelerated blood flow signals, vascular opacities, accumulation of inflammatory cells, and cartilage erosion of the rats' ankle joints to varying degrees. TGMXD-208 therapy markedly reduced serum levels of IL-1β, IL-6, and TNF-α compared to the model group. Besides, it also reduced organ indices. Furthermore, TGMXD-208 diminished the protein levels of p-PI3K and p-AKT. However, hematological parameters were not considerably impacted by TGMXD-208, and no appreciable aberrant alterations were noted in the organs.

**Discussion:**

TGMXD-208 alleviates RA pathology by suppressing PI3K/AKT pathway activation and reducing the levels of pro-inflammatory cytokines. Its dual PI3Kδ/γ targeting mitigates synovial hyperplasia and cartilage erosion in AA rats. Notably, 5 mg/kg TGMXD-208 demonstrated safety with no hematological or organ toxicity, supporting its therapeutic potential for RA

**Conclusion:**

TGMXD-208 cuts down arthritic symptoms in AA rats by blocking the PI3K/AKT pathway, making it a potential treatment option for RA.

## 1 INTRODUCTION

The pathological hallmarks of Rheumatoid Arthritis (RA), a chronic inflammatory disease, include the development of vascular opacities, cartilage degradation, and the growth of synovial tissue in the joints [1]. The pathogenesis and etiology of RA remain largely elusive. Glucocorticosteroids, biologics, and anti-rheumatic medications are the main clinical treatments for RA, which currently has no known cure. However, these drugs often exhibit significant adverse effects [2]. Consequently, it is imperative to explore potential pathogenesis and therapeutic agents for RA.

The pathogenesis of RA is associated with the aberrant transduction of multiple signaling pathways, notably the PI3K/AKT pathway. Activation of this pathway enhances chondrocyte proliferation and bone degradation while suppressing apoptosis and autophagy [3]. Moreover, the PI3K/AKT pathway exhibits hyperactivation in conditions, such as tumors, osteoarthritis, and diabetes [4-6]. Various factors and cytokines, including insulin and glucose, can trigger this pathway [7]. When tyrosine kinases and G protein-coupled receptors are stimulated by these molecules, the P85 regulatory subunit of PI3K relocates near the plasma membrane. Here, the P110 subunit associates with the P85 subunit, converting the substrate phosphatidylinositol-4,5-bisphosphate (PIP2) to phosphatidylinositol-3,4,5-trisphosphate (PIP3) [8]. PIP3 serves as a secondary messenger, binding to the Pleckstrin Homology (PH) domain and drawing Akt to the plasma membrane. Once activated, Akt phosphorylates numerous kinases, influencing a range of cellular processes, including cell activation and proliferation [9]. PI3K, a lipid kinase, specifically catalyzes phosphatidylinositol within the cell's cytoplasm and exists in three distinct types: I, II, and III [10]. Current research focuses mostly on type I PI3K, a heterodimeric protein with two components: the regulatory subunit (P85) and the catalytic subunit (P110). Class I PI3K is additionally divided into two subclasses: class IA and IB. Notably, class IA PI3K is segmented into three isoforms: PI3K α, β, and δ. These are made up of the p85 regulatory subunits and the catalytic subunits p110α, p110β, or p110δ. The p85 regulatory subunit can be activated *via* protein tyrosine kinase signaling. On the other hand, class IB PI3K encompasses the PI3Kγ isoform, which consists of the p110γ catalytic subunit and the p85 regulatory subunit. This subunit can be triggered by G protein-coupled receptor signaling [11].

TGMXD-208 is a newly developed dual PI3Kδ/γ inhibitor, distinguished by its unique seven-membered spirocyclic spacer between the quinazolone template and the diaminopyrimidine-5-carbonitrile fragment [12]. Unlike the other two isoforms of class I PI3K, which are ubiquitously distributed, PI3Kγ and PI3Kδ are primarily expressed in leukocytes [13]. PI3Kγ is abundantly expressed in myeloid cells, where it enhances chemotaxis and Reactive Oxygen Species (ROS) production by neutrophils [14]. Conversely, PI3Kδ is crucial for maintaining B cell homeostasis and function, and it is indispensable for thymic pre-T cell development [15, 16]. The inhibition of PI3Kγ effectively suppresses neutrophil chemotaxis, thereby inhibiting the progression of joint inflammation and cartilage erosion [17]. A deficiency in PI3Kδ results in a reduction of B-cells and a diminished immune response in mice.

Furthermore, PI3Kδ regulates T-cell activity within and outside lymph nodes and also controls T-cell recruitment and retention in inflammatory tissues [18]. Given the expression patterns of PI3Kδ and PI3Kγ, inhibitors, such as TGMXD-208, that target both may offer a promising therapeutic approach for treating RA. Notably, due to its specificity for PI3Kγ and PI3Kδ, TGMXD-208 can circumvent adverse effects like gastrointestinal reactions, skin rashes, and hyperglycemia, which are associated with pan-PI3K inhibitors that block all four class I PI3K isoforms [19].

This study aimed to determine if TGMXD-208 demonstrates anti-rheumatoid arthritis activity by inhibiting the PI3K/AKT signaling pathway using a CFA-stimulated Adjuvant-induced Arthritis (AA) rat model. The findings could pave the way for innovative RA treatment strategies.

## 2 MATERIALS AND METHODS

### 2.1 Chemicals and Antibodies

TGMXD-208 was synthesized in-house by the School of Pharmacy at Anhui University of Traditional Chinese Medicine (Patent No.: CN 201910709742.4; Structural formula: as depicted in Fig. (**1A**); English name: (S)-2,4-diamino-6-(6-(5-chloro-4-oxo-3-phenyl-3,4-dihydroquinazolin-2-yl)-5-azaspiro [2.^4^] heptan-5-yl) pyrimidine-5-carbonitrile)) [20]. Tonghua Maoxiang Pharmaceutical Co., Ltd. (Tonghua, China) was the supplier of methotrexate (MTX). Sodium carboxymethyl cellulose (CMC-Na) was sourced from Shanghai Qiangshun Chemical Reagent Co., Ltd. (Shanghai, China). The enzyme-linked immunosorbent assay kit for rat TNF-α, IL-6, and IL-10 was purchased from MultiSciences(Lianke)Biotech Co., Ltd. (Zhejiang, China). Antibodies for PI3K, Akt, p-Akt, p-PI3K, GAPDH, and normal rabbit IgG were procured from Wuhan Mitaka Biotechnology Co., Ltd. (Wuhan, China).

### 2.2 Animals

From Jinan Pan Yue Laboratory Animal Breeding Co. (SCXK (Lu) 20190003, China), male Sprague-Dawley (SD) rats aged 6–8 weeks with initial body weights of 180–220 g were acquired. All animals had unrestricted access to standard laboratory food and water while being kept in standard circumstances, which included 25 ± 2 °C, 60 ± 5% humidity, and a 12-hour light/12-hour dark cycle. The Laboratory Animal Ethics Committee of Anhui University of Traditional Chinese Medicine authorized all experimental protocols, and they were carried out in compliance with the AHUCM-rats-2020216 guidelines for the care and use of animals. All animals were given pentobarbital anesthesia before being put to death *via* carbon dioxide asphyxiation and bleeding in order to reduce the amount of agony they endured.

### 2.3 Induction of AA

Pharmacological studies were conducted on AA rats, which were randomly assigned to one of six experimental groups (n = 6 each): a model group, three TGMXD-208 groups (1.25 mg/kg, 2.5 mg/kg, and 5 mg/kg), an LY294002 group (1.2 mg/kg), and an MTX group (0.5 mg/kg). Additionally, there was a normal control group consisting of six animals. Drug suspensions were formulated using 0.5% carboxymethyl cellulose sodium (CMC-Na). LY294002, a well-studied pan-PI3K inhibitor, has been instrumental in elucidating PI3K function. Building on prior research, we induced AA in an animal model by administering a subcutaneous injection of CFA into the right hind paw on day 1. This was followed by daily oral administration of TGMXD-208 or intraperitoneal injection of LY294002 from day 16 to 29, depending on the group, with MTX being administered orally every three days. For the same amount of time, rats in the AA group were given a comparable volume of 0.5% CMC-Na.

### 2.4 Arthritis Score

The assessment of arthritis was conducted by two independent observers who were not privy to the experimental protocol. Prior to modeling, the volume of the left hindfoot was gauged using a PV200 volumetric instrument. After modeling was effective, the left hindfoot's volume was checked every three days. The calculation for foot-plantar swelling was determined by subtracting the pre-immunization foot-plantar swelling from the post-immunization foot-plantar swelling. Joint swelling was meticulously documented at three-day intervals, commencing from the 14^th^ day post-immunization, and subsequently scored utilizing the arthritis index (Table **1**). The arthritis index (AI) was quantitatively assessed in AA rats at various time points subsequent to immunization [21].

### 2.5 Organ Index

The rats in all of the groups were weighed before the experiment was over. Their spleen and thymus were then removed, weighed, and documented. The spleen index (spleen weight/body weight) and thymus index (thymus weight/body weight) were finally calculated.

### 2.6 X-ray Analysis

A 4% paraformaldehyde solution was used to immobilize the rats' ankle joints. The samples were then exposed to X-ray irradiation in an animal irradiator with a dosage of 0.1 mGy and particular parameters, including an image resolution of 15LP/min and photographic settings of 22.0 KV and 4 mA. Changes in the joint space, joint distortion, and soft tissue edema were noted.

### 2.7 Hematoxylin Eosin Stain

The knee joints of rats used in pharmacological studies were removed, preserved for 72 hours in 4% paraformaldehyde, calcified for 20 days in 10% EDTA-2Na, then dried and made clear. These samples were embedded in 21 blocks of paraffin wax, sectioned at 5 μm thickness, and the resulting paraffin sections were stained with hematoxylin and eosin (HE). Rats used in toxicological experiments also had their heart, liver, spleen, lungs, and kidneys removed, fixed in paraformaldehyde for 72 hours, calcified using 10% EDTA-2Na, dehydrated, and rendered translucent. These organs were sectioned at 5 μm, embedded in 60 blocks of paraffin wax, and stained with HE.

### 2.8 Extraction of Synovial Tissue

The knee joints of the euthanized rats were carefully detached from the base of the thighs, treated with Neosporin, and transferred to a sterile workbench. A longitudinal incision was made in the skin to reveal the knee joint and its cavity, where a smooth, light yellow synovial tissue became apparent. Using ophthalmic forceps, this synovial tissue was delicately clamped and subsequently excised with precision. The extracted tissue was rinsed with PBS; a portion was preserved at -80°C, while the remainder was cultured in flasks. Primary cells extracted from synovial tissue were grown in DMEM media containing 20% fetal bovine serum. For subsequent experiments, Fibroblast-like Synoviocytes (FLS) from the 3rd to 5th generations were utilized [22].

### 2.9 Analysis of Rat Serum Cytokines

After the rats were put to sleep, blood was extracted from their abdominal aortas and left to stand for an hour at ambient temperature. The serum was subsequently centrifuged at 5000 revolutions per minute for 15 minutes, then separated and stored at -80°C. Blood was drawn from the abdominal aortas of rats and allowed to stand at room temperature for one hour. Following the manufacturer's instructions, ELISA kits were used to measure the concentrations of TNF-α, IL-6, and IL-1β.

### 2.10 Immunohistochemistry

Rat knee joint tissues were processed using paraffin embedding. The phosphorylation concentrations of PI3K and AKT in these tissues were determined using anti-Rat p-PI3K and p-AKT antibodies. Positive expression was identified by a brown or yellow coloration. The expression rates of p-PI3K and p-AKT proteins in the knee joints of seven rat groups were subsequently measured.

### 2.11 Western Blot Analysis

For the Western blot experiments, we utilized 3 to 5 generations of FLS. The protein contents were pulled out with RIPA lysis buffer, while nuclear proteins were isolated following the instructions in the Nuclear Protein Extraction Kit. The protein content was measured with a BCA kit. Aliquots of the protein samples were then added to each well for SDS-PAGE before being transferred to PVDF membranes. Following this, the membrane was sealed with 5% skimmed milk for 2 hours at room temperature. Subsequently, the membrane was incubated with GAPDH, PI3K, AKT, p-PI3K, and p-AKT overnight at a temperature of 4°C, followed by the secondary antibody for another 2 hours at room temperature. The gray values of each band were analyzed using ImageJ software. For statistical analysis, the gray values of the target proteins were normalized using control proteins.

### 2.12 Routine Blood Analysis

Blood samples were procured from rats to conduct standard blood tests, which included the analysis of Platelet (PLT) count, Hemoglobin (HGB) levels, White Blood Cell (WBC) count, and Red Blood Cell (RBC) count.

### 2.13 Statistical Analysis

To perform the statistical assessment, GraphPad Prism V.8.0.2 (GraphPad, USA) was used. The mean ± SD was used to represent the data in the figures. A one-way ANOVA was used to determine the differences between the experimental groups. Statistical significance was defined as a *p*- value of less than 0.05.

## 3 RESULTS

### 3.1 TGMXD-208 Improves Joint Pathology in AA Rats

**Fig. (1):**
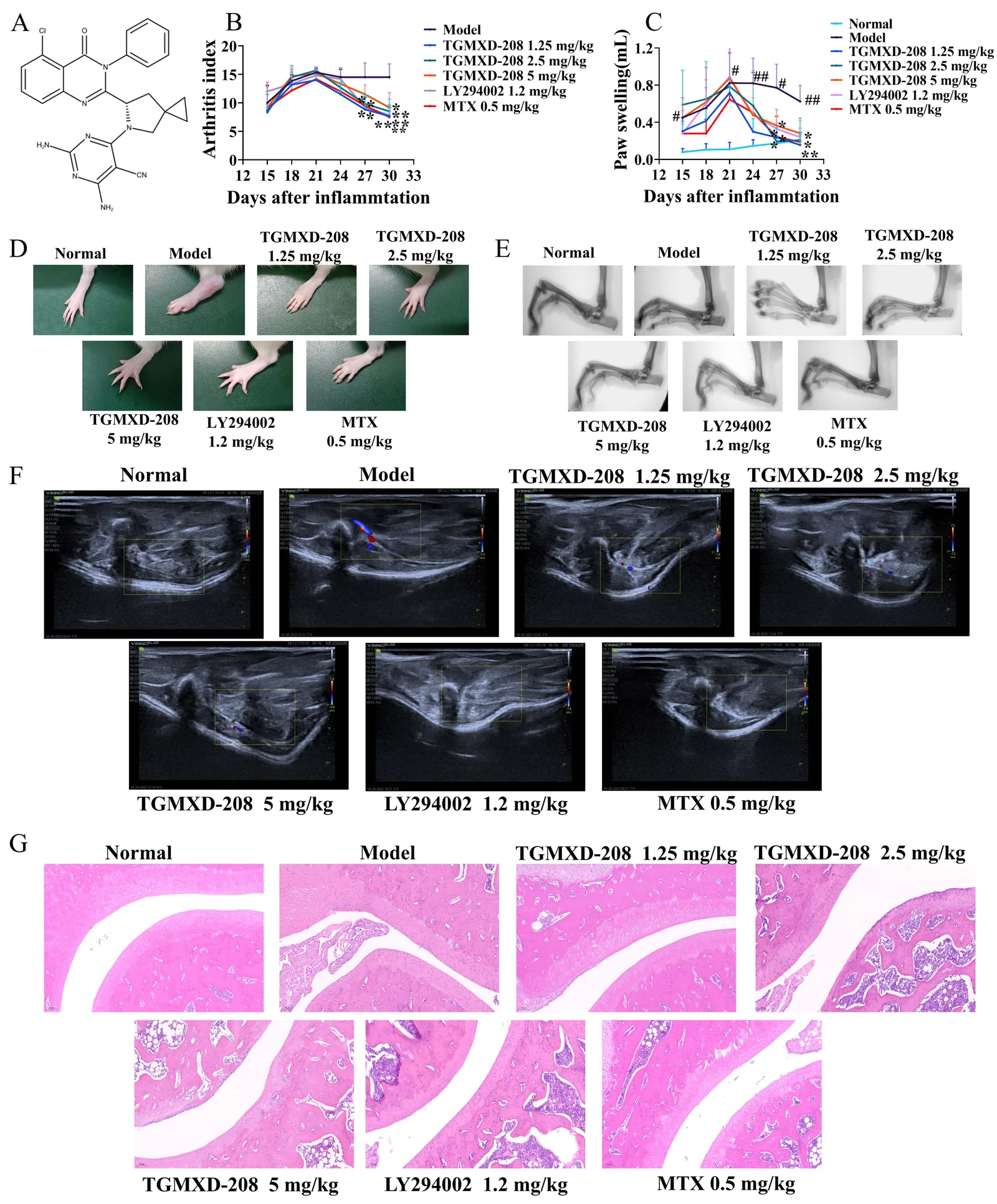
TGMXD-208 improves joint pathology in AA rats. (**A**): TGMXD-208 structure; (**B**): Arthritis index; (**C**): Paw swelling; (**D**): Representative paw images of rats; (**E**): X-ray images of the ankle joint; (**F**): blood flow signals in the knee joint; (**G**): HE staining of the knee joint (scale bar= 200 μm). The values in the normal group differ significantly from those in the ^##^*P*<0.01 group. Significant differences from the AA group's values are indicated by *P<0.05, **P<0.01. Error bars represent the standard error of the mean (SEM), with N = 6 per group and were analyzed using Repeated Measures ANOVA, followed by multiple - comparisons analysis using GraphPad Prism version 8.0.2.

A rat model of AA was developed to mimic the disease in order to investigate the effects of TGMXD-208 on RA. Following the successful establishment of the model on day 15, groups were randomly assigned. From day 16 to day 29, the AA rats were treated with varying doses of TGMXD-208 (1.25 mg/kg, 2.5 mg/kg, and 5 mg/kg), LY294002 (1.2 mg/kg), and MTX (0.5 mg/kg). AA rats exhibited pronounced redness and swelling of the toes, along with multiple joint nodules by day 29. The model group's arthritis index and level of toe inflammation both sharply rose, reaching a peak on day 21. However, the redness, nodules, and arthritis index of the rat toes in each dose group of TGMXD-208, LY294002, and MTX showed improvement over time compared to the AA group (Figs. **1B**-**D**). X-ray examination of the rats' ankle joint lesions revealed deformation and breakage in the model group, with swollen soft tissues and a smaller joint space than the normal group. Following drug administration, there was a significant improvement in joint damage. Changes in the blood flow signals in the rats' joints were picked up by the small animal ultrasound imaging system (Fig. **1E**). The ultrasound results indicated increased blood flow in the knee joints of AA rats, which returned to normal after drug administration and treatment (Fig. **1F**). Histopathological analysis revealed that the joint surfaces of the rats in the normal group were free from abnormalities, while those in the model group exhibited abnormalities, such as fragmentation of joint tissues, synovial hyperplasia, soft tissue swelling, and inflammatory cell infiltration (Fig. **1G**). Conversely, TGMXD-208, LY294002, and MTX improved these pathologies to varying degrees.

The findings presented herein indicate that TGMXD-208 ameliorates joint pathology and exerts a protective effect on rats with AA.

### 3.2 Effects of TGMXD-208 on Organ Index and Pro-Inflammatory Factors in AA Rats

**Fig. (2):**
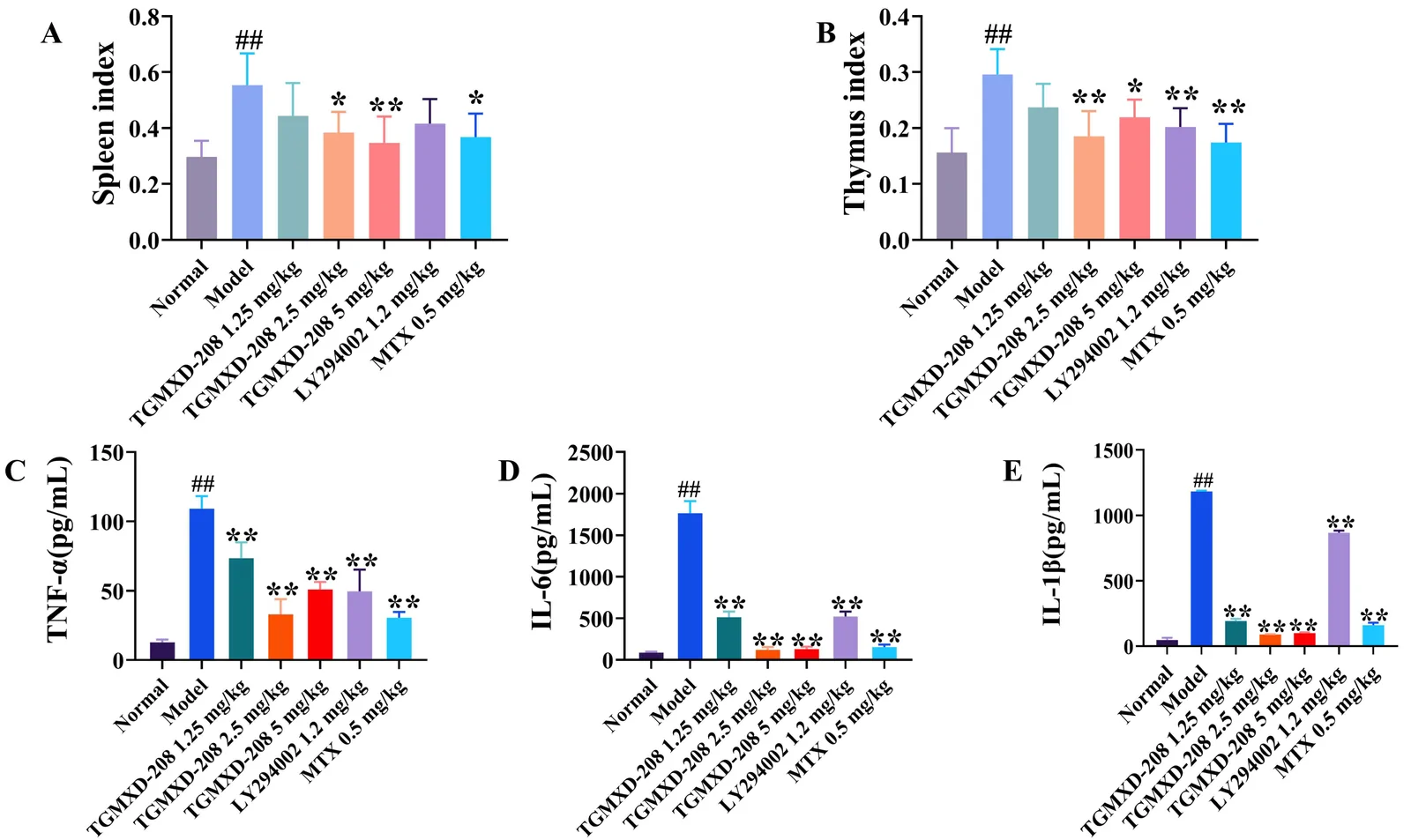
Effects of TGMXD-208 on organ index and pro-inflammatory factors in AA rats. (**A**): Spleen index; (**B**): Thymus index; (**C**): Expression of TNF-α in rat serum; (**D**): Expression of IL-6 in rat serum; (**E**): Expression of IL-1β in rat serum. The values in the normal group differ significantly from those in the ^##^*P*<0.01 group. Significant differences from the AA group's values are indicated by *P<0.05, **P<0.01. Data are mean ± SEM, N = 6, and were analyzed using one-way ANOVA, followed by Dunnett's multiple-comparisons test using GraphPad Prism version 8.0.2.

The spleen and thymus constitute the primary organs of the immune system. Indices derived from these organs, namely the spleen index and thymus index, serve as crucial indicators of the body's overall immune function. Rats in the model group had noticeably greater spleen and thymus indices than normal rats due to inflammatory development, as seen in Figs. (**2A**, **B**). However, treatment with TGMXD-208, MTX, and LY294002 resulted in a significant reduction in both the spleen and thymus indices relative to the model group. The serum levels of pro-inflammatory factors were assessed using ELISA (Figs. **2C**-**E**). The findings revealed that the expression levels of IL-1β, IL-6, and TNF-α were significantly increased in the model group rats. In contrast, animals given a dose of TGMXD-208 showed a significant drop in these inflammatory factor levels. This implies that TGMXD-208 exerts a regulatory impact on the immune function of AA rats, effectively mitigating their inflammatory response.

### 3.3 Effect of TGMXD-208 on PI3K/AKT Signaling Pathway in AA Rats

**Fig. (3):**
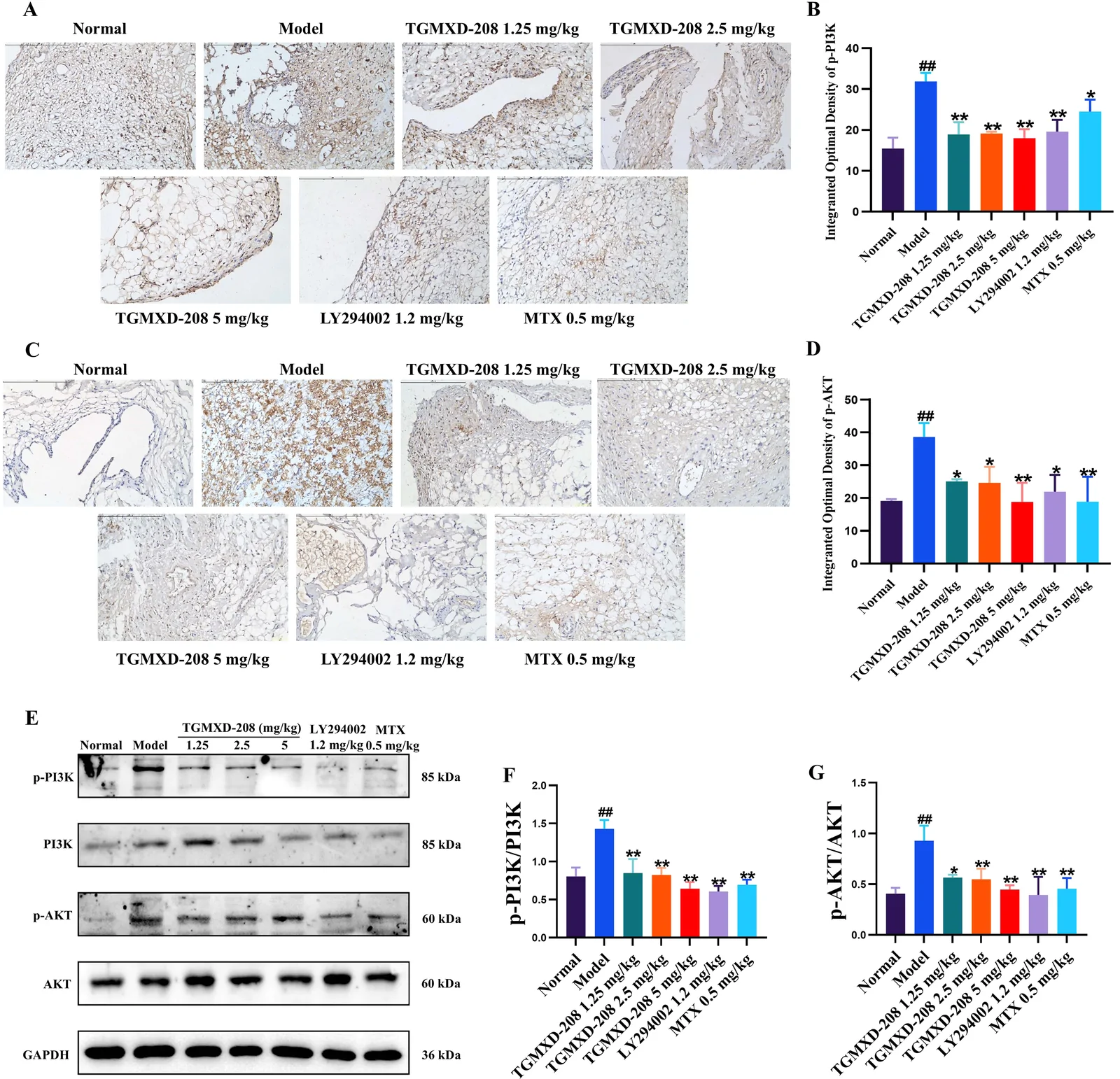
Effect of TGMXD-208 on PI3K/AKT signaling pathway in AA rats. (**A & B**): immunohistochemical expression of anti-p-PI3K (×200); (**C & D**): immunohistochemical expression of anti-p-AKT (×200); (**E**): The bands of Western blot; (**F & G**): The densitometric analysis of bands. The values in the normal group differ significantly from those in the ^##^*P*<0.01 group. Significant differences from the AA group's values are indicated by *P<0.05, **P<0.01. Data are mean ± SEM, N = 6, and were analyzed using one-way ANOVA, followed by Dunnett's multiple-comparisons test using GraphPad Prism version 8.0.2.

Our study focused on the key proteins of the PI3K/AKT signaling pathway and their phosphorylation levels to elucidate the anti-rheumatoid arthritis mechanism of TGMXD-208. We utilized immunohistochemistry to determine the amounts of p-PI3K and p-AKT proteins in rat FLS. The findings revealed a significant increase in the expression of p-PI3K and p-AKT in the model group rats, which was significantly reduced after drug administration (Figs. **3A**-**D**). To confirm these findings, we used Western blot to measure the amounts of phosphorylated PI3K and AKT proteins in rat FLS. The results indicated a significant phosphorylation of PI3K and AKT in the model group rats, which was significantly inhibited by TGMXD-208, LY294002, and MTX compared to the model group (Figs. **3E**-**G**). This implies that TGMXD-208 can considerably suppress the generation of p-PI3K and p-AKT proteins in AA rats' FLS.

### 3.4 Toxicological Evaluation of TGMXD-208 in AA Rats

**Fig. (4):**
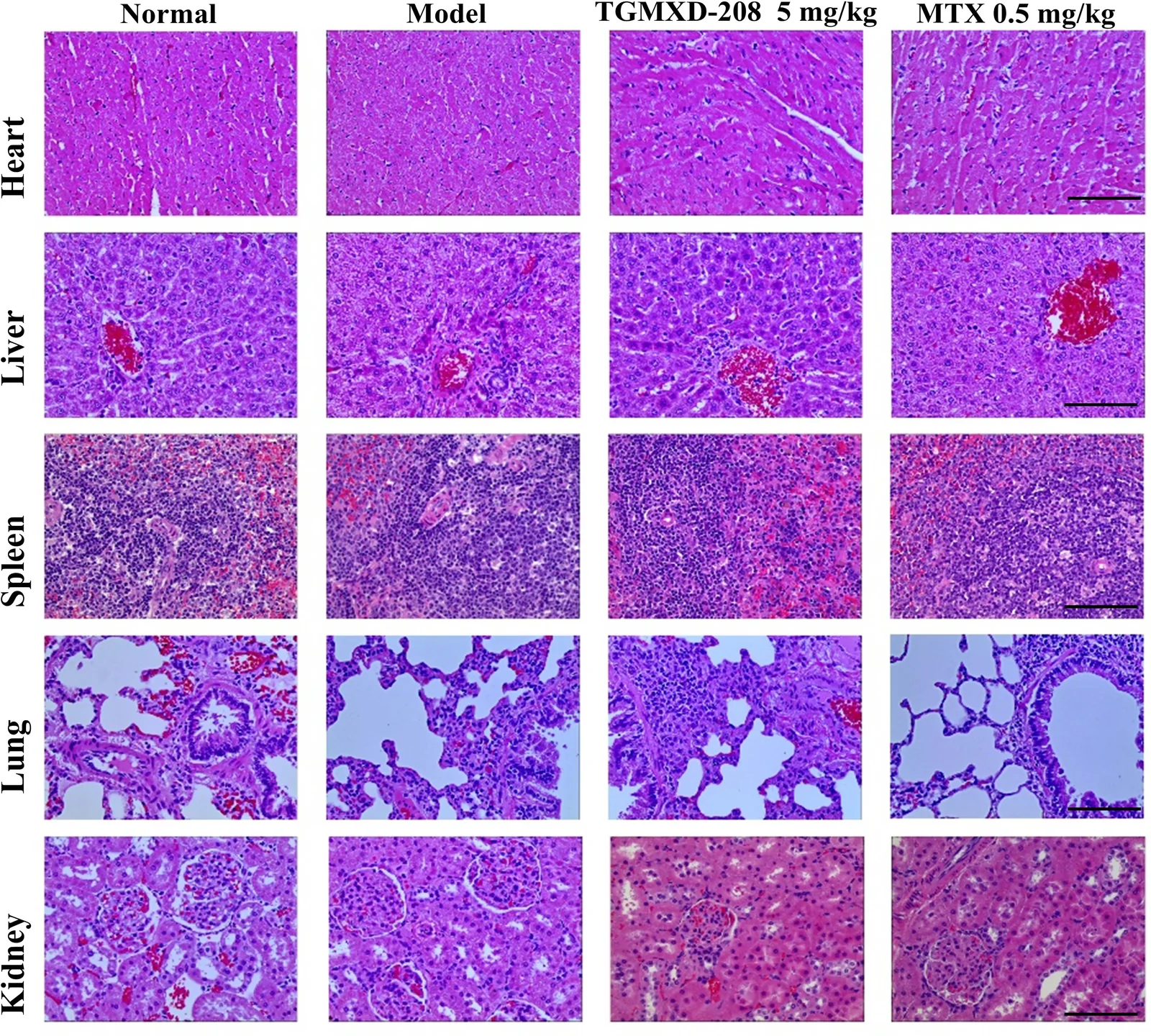
Safety of TGMXD-208 in AA rats. Histological sections stained with HE of heart, liver, spleen, lung, and kidney tissues. (scale bar= 100 μm).

This study aimed to assess the safety of TGMXD-208 on AA rats by examining their blood routine and the pathological histology of their primary organs. The blood routine results (Table **2**) revealed a significantly lower hemoglobin count and higher platelet and leukocyte counts in AA rats compared to the normal group. While there was some improvement in the administered group compared to the model group, the difference was not significant. This suggests that a dose of 5 mg/kg of TGMXD-208 does not significantly affect blood routine indices. The vital organs - heart, liver, spleen, lungs, and kidneys - were extracted from rats in each group. Visual observation found their size, color, and structure to be normal. Furthermore, histopathological section results (Fig. **4**) showed no significant abnormal changes in any group, indicating that 5 mg/kg TGMXD-208 is non-toxic and has a good safety profile in AA rats.

## 4 DISCUSSION

The importance of the PI3K/AKT signaling pathway in RA has been shown in recent research, indicating that inhibitors of this pathway may be good options for clinical use. Current investigations into PI3K inhibitors for RA treatment include PBT-6, ZSTK474, and GS9901 [23, 24]. However, while these compounds have been examined for their anti-RA efficacy, subsequent studies on their properties and safety profiles are lacking. Our group has synthesized a novel dual inhibitor of PI3Kδ/γ, TGMXD-208 (Patent No.: CN201910709742.4), which can inhibit the PI3K signaling pathway in SU-DHL-6 cells. This implies that it could be used as a possible medication to treat B-cell cancers [12]. These findings imply that TGMXD-208 could also play a therapeutic role in tumors, inflammation, and autoimmune diseases. Our study corroborates the anti-rheumatoid arthritis activity of TGMXD-208 through *in vivo* experiments.

This study confirmed that TGMXD-208 significantly alleviated the symptoms of adjuvant arthritis. Histopathological analysis revealed that TGMXD-208 mitigated cartilage erosion, joint space narrowing, and vascular opacification formation while enhancing blood flow in AA rats. These findings validate the therapeutic efficacy of TGMXD-208 in AA rats. TNF-α is a primary pro-inflammatory cytokine linked with RA, known to augment the production of inflammatory mediators in RA [25]. Concurrently, IL-6 and IL-1β function as downstream mediators of TNF-α, inducing the differentiation of Th17 cells, which are instrumental in RA joint degradation [26]. In AA rats, the blood concentrations of inflammatory cytokines (TNF-α, IL-1β, and IL-6) were significantly decreased by TGMXD-208. On cells without membrane-bound IL-6R, IL-6 binds to sIL-6R and triggers intracellular signaling known as IL-6 trans signaling through gp130. This trans-signaling is proinflammatory and activates the PI3K/AKT pathway [27]. TNF-α prompts T cells to produce macrophage colony-stimulating factor, subsequently stimulating osteoclasts to produce RANKL, thereby indirectly promoting osteoclastogenesis [28]. Inhibition of the PI3K/AKT pathway suppresses TNF-α-induced inflammation [29]. Consequently, TGMXD-208 can diminish articular cartilage damage and bone erosion by curtailing the release of inflammatory agents and modulating the PI3K/AKT signaling pathway.

A key player in the pathophysiology of RA is the PI3K/AKT signaling pathway, whose activation causes persistent synoviocyte growth and a worsening of RA symptoms. TGMXD-208 significantly reduced the phosphorylation of the protein PI3K and AKT, key proteins in the PI3K/AKT signaling pathway, in rat Fibroblast-Like Synoviocytes (FLS), according to immunocytochemistry and Western blot studies from this study. This finding implies that TGMXD-208 could alleviate the pathological manifestations in AA rats by suppressing the PI3K/AKT signaling pathway. Notably, the expression of phosphorylated PI3K was reduced post-TGMXD-208 administration, potentially due to diminished secretion of pro-inflammatory cytokines, such as IL-1β, IL-6, and TNF-α [30]. Subsequent toxicological evaluations confirmed that a dose of 5 mg/kg TGMXD-208 exhibited no significant adverse impacts on hematological profiles or major organs in AA rats, underscoring the safety and appropriateness of the administered dose in our pharmacological investigation. In summary, given its notable therapeutic potential and commendable tolerability, TGMXD-208 holds promise as an advanced anti-RA agent.

TGMXD-208 is a PI3Kδ/γ inhibitor independently developed by our team. In this study, we preliminarily explored its therapeutic effects on AA rats through the inhibition of the PI3K/AKT signaling pathway. Our findings showed alterations in both the total protein and phosphorylated protein levels of PI3K and AKT, suggesting that TGMXD-208 may exert its effects not only through phosphorylation modifications but also by influencing the expression or translation of these proteins. The main limitations of this study lie in the lack of in-depth investigation into TGMXD-208's regulatory effects on the transcriptional levels of key genes in the PI3K/AKT signaling pathway, as well as insufficient experimental evidence regarding its impact on immune cell subsets (B cells, T cells, and neutrophils). Furthermore, the diversity of experimental models requires expansion. Future studies should address these aspects and employ multi-species animal models for validation, which would contribute to systematically elucidating TGMXD-208's anti-RA mechanisms and thereby provide a more robust theoretical foundation for developing novel RA therapeutics.

## CONCLUSION

Our research has shown that TGMXD-208 can exert a protective effect in rats with AA by inhibiting the PI3K/AKT signaling pathway. This was primarily observed through changes in joint morphology and histology in the AA rats. Furthermore, a dosage of 5 mg/kg of TGMXD-208 demonstrated a favorable safety profile in these rats. As a result, our research points to TGMXD-208 as a possible therapeutic option for RA treatment.

## Figures and Tables

**Table 1:** Arthritis index chart.

**Symptom**	**Score**
Normal	0
Erythema and Mild Ankle Joint Edema	1
Erythema and Mild Ankle-to-Toe Joint Enlargement	2
Erythema and Moderate Ankle-to-Toe Joint Swelling	3
Erythema and Significant Ankle-to-Toe Joint Swelling	4

**Table 2:** Effect of TGMXD-208 on routine blood indexes in AA rats.

-	**RBC(×10^12^/L)**	**HGB (g/L)**	**PLT (×10^9^/L)**	**WBC(×10^9^/L)**
Normal	8.43±0.42	155.14±9.28	835.4±111.03	14.59±2.27
Model	8.41±0.55	133.5±7.63**	1161.25±74.45**	20.2±2.54*
TGMXD-208 (5 mg/kg)	8.57±0.48	137.33±15.24	1041.17±214.99	16.17±3.66

**Note: ** Significant differences from the AA group's values are indicated by *P<0.05, **P<0.01. Data are mean ± SEM, N = 6, and were analyzed using one-way ANOVA, followed by Dunnett's multiple-comparisons test using GraphPad Prism version 8.0.2.

## Data Availability

The data used in this study are available within the article.

## LIST OF ABBREVIATIONS

AA: Adjuvant-induced Arthritis
IL-1β: IL-1β Interleukin-1 Beta
RA: Rheumatoid Arthritis
IL-10: Interleukin-10
PI3K: Phosphoinositide 3-kinase
TNF-α: Tumor Necrosis Factor-α
AKT: Protein Kinase B
PBS: Phosphate Buffered Saline
CFA: Complete Freund's Adjuvant
IHC: Immunohistochemistry
ELISA: Enzyme-Linked Immunosorbent Assay
HE: Hematoxylin-eosin
PLT: Platelets
RBC: Red Blood Cells
HGB: Hemoglobin
MTX: Methotrexate
WBC: White Blood Cell
PIP2: Phosphatidylinositol-4,5-bisphosphate
PIP3: Phosphatidylinositol-3,4,5-trisphosphate
PH: Pleckstrin Homology

## References

[1] Fleischmann R., Kremer J., Cush J., Schulze-Koops H., Connell C.A., Bradley J.D., Gruben D., Wallenstein G.V., Zwillich S.H., Kanik K.S. (2012). Placebo-controlled trial of tofacitinib monotherapy in rheumatoid arthritis.. N. Engl. J. Med..

[2] Liu C., He L., Wang J., Wang Q., Sun C., Li Y., Jia K., Wang J., Xu T., Ming R., Wang Q., Lin N. (2020). Anti-angiogenic effect of Shikonin in rheumatoid arthritis by downregulating PI3K/AKT and MAPKs signaling pathways.. J. Ethnopharmacol..

[3] Xue J.F., Shi Z.M., Zou J., Li X.L. (2017). Inhibition of PI3K/AKT/mTOR signaling pathway promotes autophagy of articular chondrocytes and attenuates inflammatory response in rats with osteoarthritis.. Biomed. Pharmacother..

[4] Yu L., Wei J., Liu P. (2022). Attacking the PI3K/Akt/mTOR signaling pathway for targeted therapeutic treatment in human cancer.. Semin. Cancer Biol..

[5] Zhao C., Li X., Sun G., Liu P., Kong K., Chen X., Yang F., Wang X. (2022). CircFOXO3 protects against osteoarthritis by targeting its parental gene FOXO3 and activating PI3K/AKT-mediated autophagy.. Cell Death Dis..

[6] Shum M., Bellmann K., St-Pierre P., Marette A. (2016). Pharmacological inhibition of S6K1 increases glucose metabolism and Akt signalling *in vitro* and in diet-induced obese mice.. Diabetologia.

[7] Engelman J.A., Luo J., Cantley L.C. (2006). The evolution of phosphatidylinositol 3-kinases as regulators of growth and metabolism.. Nat. Rev. Genet..

[8] Pirola L., Zvelebil M.J., Bulgarelli-Leva G., Van Obberghen E., Waterfield M.D., Wymann M.P. (2001). Activation loop sequences confer substrate specificity to phosphoinositide 3-kinase alpha (PI3Kalpha ). Functions of lipid kinase-deficient PI3Kalpha in signaling.. J. Biol. Chem..

[9] Haruta K., Mori S., Tamura N., Sasaki A., Nagamine M., Yaguchi S., Kamachi F., Enami J., Kobayashi S., Yamori T., Takasaki Y. (2012). Inhibitory effects of ZSTK474, a phosphatidylinositol 3-kinase inhibitor, on adjuvant-induced arthritis in rats.. Inflamm. Res..

[10] Markman B., Dienstmann R., Tabernero J. (2010). Targeting the PI3K/Akt/mTOR pathway-beyond rapalogs.. Oncotarget.

[11] Kong D., Yamori T. (2009). Advances in development of phosphatidylinositol 3-kinase inhibitors.. Curr. Med. Chem..

[12] Tao Q., Chen Y., Liang X., Hu Y., Li J., Fang F., Wang H., Meng C., Liang J., Ma X., Gui S. (2020). Structurally novel PI3Kδ/γ dual inhibitors characterized by a seven-membered spirocyclic spacer: The SARs investigation and PK evaluation.. Eur. J. Med. Chem..

[13] Lucas C.L., Chandra A., Nejentsev S., Condliffe A.M., Okkenhaug K. (2016). PI3Kδ and primary immunodeficiencies.. Nat. Rev. Immunol..

[14] Hawkins P.T., Stephens L.R. (2015). PI3K signalling in inflammation.. Biochim. Biophys. Acta Mol. Cell Biol. Lipids.

[15] Clayton E., Bardi G., Bell S.E., Chantry D., Downes C.P., Gray A., Humphries L.A., Rawlings D., Reynolds H., Vigorito E., Turner M. (2002). A crucial role for the p110delta subunit of phosphatidylinositol 3-kinase in B cell development and activation.. J. Exp. Med..

[16] Webb L.M.C., Vigorito E., Wymann M.P., Hirsch E., Turner M. (2005). Cutting edge: T cell development requires the combined activities of the p110γ and p110δ catalytic isoforms of phosphatidylinositol 3-kinase.. J. Immunol..

[17] Randis T.M., Puri K.D., Zhou H., Diacovo T.G. (2008). Role of PI3Kδ and PI3Kγ in inflammatory arthritis and tissue localization of neutrophils.. Eur. J. Immunol..

[18] Johansen K.H., Golec D.P., Thomsen J.H., Schwartzberg P.L., Okkenhaug K. (2021). PI3K in T cell adhesion and trafficking.. Front. Immunol..

[19] Patel L., Chandrasekhar J., Evarts J., Forseth K., Haran A.C., Ip C., Kashishian A., Kim M., Koditek D., Koppenol S., Lad L., Lepist E.I., McGrath M.E., Perreault S., Puri K.D., Villaseñor A.G., Somoza J.R., Steiner B.H., Therrien J., Treiberg J., Phillips G. (2016). Discovery of orally efficacious phosphoinositide 3-Kinase δ inhibitors with improved metabolic stability.. J. Med. Chem..

[20] Ma X. (2019). A class of PI3Kδ inhibitors and their uses..

[21] Chen S., Wang J., Wang J., Jia X., Xuan Z., Cheng Z., Meng X., Su W. (2023). Wnt/β-catenin signaling pathway promotes abnormal activation of fibroblast-like synoviocytes and angiogenesis in rheumatoid arthritis and the intervention of Er Miao San.. Phytomedicine.

[22] Fang W. (2016). The effects of BAFF and its receptors signal on dendritic cells and fibroblast-like synoviocytes in experimental arthritis and the therapeutic effects of CP-25.. PhD Thesis, Anhui Medical University.

[23] Kim J., Jung K.H., Yoo J., Park J.H., Yan H.H., Fang Z., Lim J.H., Kwon S.R., Kim M.K., Park H.J., Hong S.S. (2020). PBT-6, A novel PI3KC2Γ inhibitor in rheumatoid arthritis.. Biomol. Ther..

[24] Toyama S., Tamura N., Haruta K., Karakida T., Mori S., Watanabe T., Yamori T., Takasaki Y. (2010). Inhibitory effects of ZSTK474, a novel phosphoinositide 3-kinase inhibitor, on osteoclasts and collagen-induced arthritis in mice.. Arthritis Res. Ther..

[25] Kapoor M., Martel-Pelletier J., Lajeunesse D., Pelletier J.P., Fahmi H. (2011). Role of proinflammatory cytokines in the pathophysiology of osteoarthritis.. Nat. Rev. Rheumatol..

[26] Zheng Y., Sun L., Jiang T., Zhang D., He D., Nie H. (2014). TNFα promotes Th17 cell differentiation through IL-6 and IL-1β produced by monocytes in rheumatoid arthritis.. J. Immunol. Res..

[27] Zegeye M.M., Lindkvist M., Fälker K., Kumawat A.K., Paramel G., Grenegård M., Sirsjö A., Ljungberg L.U. (2018). Activation of the JAK/STAT3 and PI3K/AKT pathways are crucial for IL-6 trans-signaling-mediated pro-inflammatory response in human vascular endothelial cells.. Cell Commun. Signal..

[28] Hayer S., Pundt N., Peters M.A., Wunrau C., Kühnel I., Neugebauer K., Strietholt S., Zwerina J., Korb A., Penninger J., Joosten L.A.B., Gay S., Rückle T., Schett G., Pap T. (2009). PI3Kγ regulates cartilage damage in chronic inflammatory arthritis.. FASEB J..

[29] Tang Z.L., Zhang K., Lv S.C., Xu G.W., Zhang J.F., Jia H.Y. (2021). LncRNA MEG3 suppresses PI3K/AKT/mTOR signalling pathway to enhance autophagy and inhibit inflammation in TNF-α-treated keratinocytes and psoriatic mice.. Cytokine.

[30] Hao F., Wang Q., Liu L., Wu L.B., Cai R.L., Sang J.J., Hu J., Wang J., Yu Q., He L., Shen Y.C., Miao Y.M., Hu L., Wu Z.J. (2022). Effect of moxibustion on autophagy and the inflammatory response of synovial cells in rheumatoid arthritis model rat.. J. Tradit. Chin. Med..

